# Effects of low dose estrogen therapy on the vaginal microbiomes of women with atrophic vaginitis

**DOI:** 10.1038/srep24380

**Published:** 2016-04-22

**Authors:** Jian Shen, Ning Song, Christopher J. Williams, Celeste J. Brown, Zheng Yan, Chen Xu, Larry J. Forney

**Affiliations:** 1 Department of Anatomy, Histology and Embryology, Shanghai Jiao Tong University School of Medicine, Shanghai, China, 200025; 2 Shanghai Key Laboratory of Reproductive Medicine, Shanghai, China, 200025; 3 Departments of Statistical Science, University of Idaho, Moscow, ID, 83844, Russia; 4 Biological Sciences, University of Idaho, Moscow, ID, 83844, Russia; 5 Institute for Bioinformatics and Evolutionary Studies (IBEST), University of Idaho, Moscow, ID, 83844, Russia; 6 Department of Obstetrics and Gynecology, Ruijin Hospital, Shanghai Jiao Tong University School of Medicine, Shanghai, China, 200025; 7 Reproductive Medicine Center, Shanghai Ninth Hospital, Shanghai Jiao Tong University School of Medicine, Shanghai, China, 200025

## Abstract

Atrophic vaginitis (AV) is common in postmenopausal women, but its causes are not well understood. The symptoms, which include vaginal itching, burning, dryness, irritation, and dyspareunia, can usually be alleviated by low doses of estrogen given orally or locally. Regrettably, the composition of vaginal bacterial communities in women with AV have not been fully characterized and little is known as to how these communities change over time in response to hormonal therapy. In the present intervention study we determined the response of vaginal bacterial communities in postmenopausal women with AV to low-dose estrogen therapy. The changes in community composition in response to hormonal therapy were rapid and typified by significant increases in the relative abundance of *Lactobacillus* spp. that were mirrored by a decreased relative abundance of *Gardnerella*. These changes were paralleled by a significant four-fold increase in serum estradiol levels and decreased vaginal pH, as well as nearly a two-fold increase in the Vaginal Maturation Index (VMI). The results suggest that after menopause a vaginal microbiota dominated by species of *Lactobacillus* may have a beneficial role in the maintenance of health and these findings that could lead to new strategies to protect postmenopausal women from AV.

Atrophic vaginitis (AV) is a common affliction that develops in 25% to 50% of postmenopausal women^1^. Its symptoms include vaginal itching, burning, dryness, irritation, and dyspareunia, which degrade a woman’s quality of life and are unlikely to diminish without treatment^2^. Studies have shown that AV is associated with estrogen deficiencies during menopause that cause reduced vaginal secretions, vulvovaginal atrophy, and decreased glycogen production by vaginal epithelial cells. It is accompanied by decreased numbers of lactobacilli and lactic acid production, causing an increased vaginal pH that possibly renders the vagina more susceptible to infections^1^,^2^. Although the occurrence of vulvovaginal atrophy and increased vaginal pH are nearly universal in menopause, most elderly women do not present with genital complaints, which suggests there are unknown factors that distinguish AV patients and asymptomatic women. So far the preferred treatment of AV symptoms is low-dose estrogen therapy, which minimizes the risk of endometrial and breast cancers that are responsive to estrogen^3^,^4^,^5^. However, the effects of this therapy on the vaginal microbiome are not well understood.

The vaginal microbiome and host immunity are two components of a mutualistic relationship that play a pivotal role in maintaining health and minimizing risk to adverse urogenital problems such as AV. In reproductive age women the vaginal microbiome is often dominated by various species of *Lactobacillus* that occur in high numbers. This is widely thought to be a normal and healthy state. In contrast, communities in which lactobacilli are supplanted by any of various strictly anaerobic bacteria are often accompanied by symptoms of bacterial vaginosis (BV) that places these women at higher risk to disease as well as obstetric and gynecological complications^6^. Although considerable efforts have been made to better understand these communities in reproductive-age women, far less is known about the composition and function of these communities in women after menopause.

In this study we compared the vaginal microbiota of healthy post-menopausal women (H group) to those of post-menopausal women with atrophic vaginitis (AV group). Women in the AV group were given low-dose estrogen therapy and followed over a four-week treatment period to observe the response of their vaginal microbiota to the treatment. These were compared to the vaginal microbiota of the untreated H group over the same period of time.

## Results

Sixty of the 67 women originally enrolled completed this longitudinal study. Reasons for not continuing in the study included: loss to follow-up (N = 3 in H group), adverse events (nausea in one subject of the AV group), non-compliance (one subject of the AV group), and other reasons (N = 2, one from each group). The microbial community data from samples of one subject (subject 20) in the H group were not included in data analysis because the numbers of DNA sequence reads from vaginal samples were too low. Finally, 59 postmenopausal women with (N = 30) or without (N = 29) AV were enrolled in the present study. Overall, there were no major differences in the baseline characteristics of women in the two groups (Table 1). Subjects had a mean age of 56 years and the range of time since last menses was 21–118 months.

The species composition and structure of vaginal bacterial communities were determined by classifying 16S rRNA gene sequences recovered from mid-vaginal swab samples collected by a physician at 0, 2 and 4 weeks. DNA sequencing using barcoded primers (Supplementary Table S1) produced a dataset that consisted of 982,704 high-quality 16S rRNA gene sequences from 177 samples. On average 5459 ± 2345 (SD) reads were obtained from each sample and these were classified using the Ribosomal Database Project Naïve Bayesian Classifier^7^. Overall, a total of 288 taxa were observed in the vaginal microbiota of women in the two groups (Supplementary Table S2). The taxonomic assignments of vaginal bacterial community members and the associated metadata for each subject are shown in Supplementary Table S3.

### Characterization of vaginal bacterial communities of healthy post-menopausal women

Contrary to the common wisdom that *Lactobacillus* spp. are usually absent from the vaginal microbiome after menopause^8^, we found that lactobacilli were prominent members of vaginal communities of most healthy post-menopausal women. They constituted more than half of communities in 55.2% of the women enrolled in the study and more than 0.1% of all bacteria in 83% of the subjects. Other bacteria such as *Gardnerella*, *Lactobacillales*, *Prevotella* and *Atopobium* were also common and found in 82.8%, 79.3%, 75.9% and 65.5% of communities, respectively, but on average occurred in lower proportions (16.7%, 0.7%, 7.4% and 2.7%, respectively).

**Figure 1:**
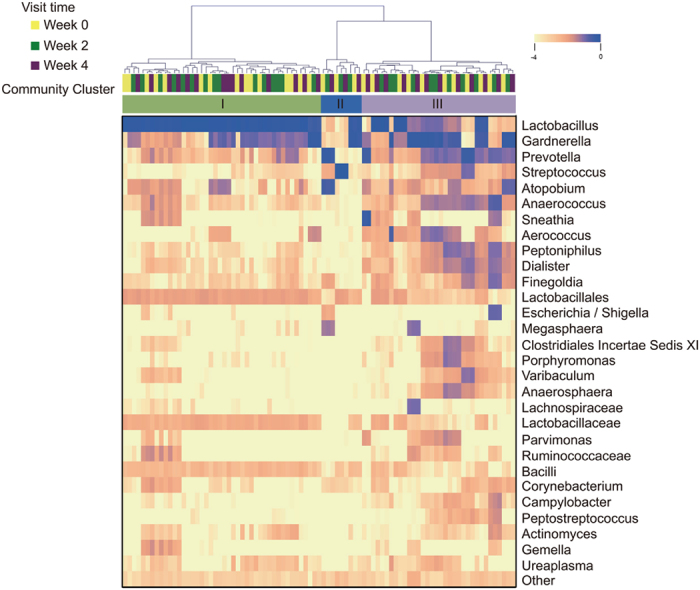
Hierarchical clustering of vaginal microbial communities in healthy postmenopausal women and a heat map showing the log_10_-transformed proportions of bacterial genera in each community. The visit time of each sample is indicated by the color-coded bar immediately below the dendrogram. Analysis of similarities in community composition and structure resulted in three clusters as indicated in the color bar above the heatmap.

The vaginal bacterial communities of healthy post-menopausal women sampled at three visits (weeks 0, 2 and 4 weeks) were clustered based on differences in species composition and their relative abundances (see Fig. 1 and Supplemental Material). We identified three major clusters of vaginal community types. Communities of cluster I were most common and present in 50.6% of the samples. These communities were dominated by *Lactobacillus* that constituted >50% of all sequences obtained. The communities of cluster II were present in 10.3% of the samples and had high proportions of *Prevotella*, *Atopobium*, *Streptococcus* or *Gardnerella*. The remainder of communities in Cluster III (39.1%) contained appreciable proportions of various species of strictly anaerobic bacteria, including *Gardnerella*, *Prevotella*, *Streptococcus*, *Atopobium*, *Anaerococcus*, *Sneathia*, *Aerococcus*, *Peptoniphilus*, *Dialister* and *Finegoldia* along with lower proportions of *Lactobacillus*. Even though many of these bacterial taxa, including *Gardnerella*, *Prevotella*, *Atopobium* and *Sneathia* have previously been associated with BV, none of these women had clinical symptoms associated with BV.

**Figure 2:**
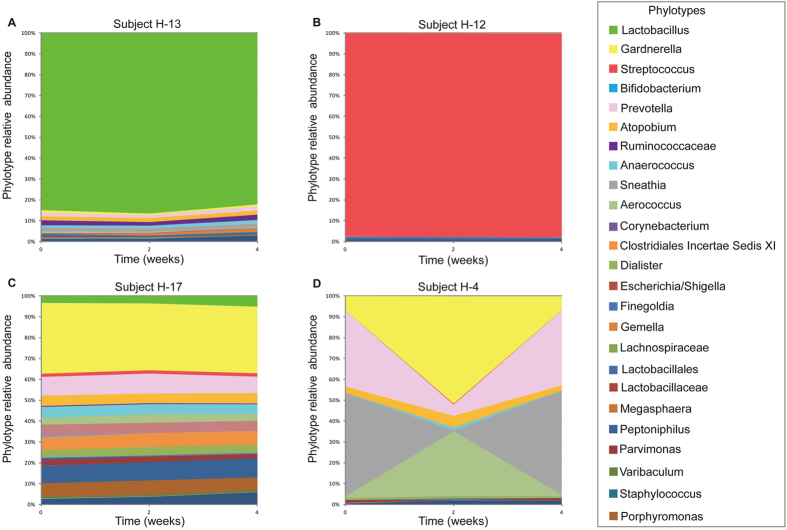
Interpolated bar plots of phylotype relative abundances in four selected subjects in the H group over 4 weeks (panels **A–D**). Color key for each phylotype represented in the interpolated bar plots are on the right side.

**Figure 3:**
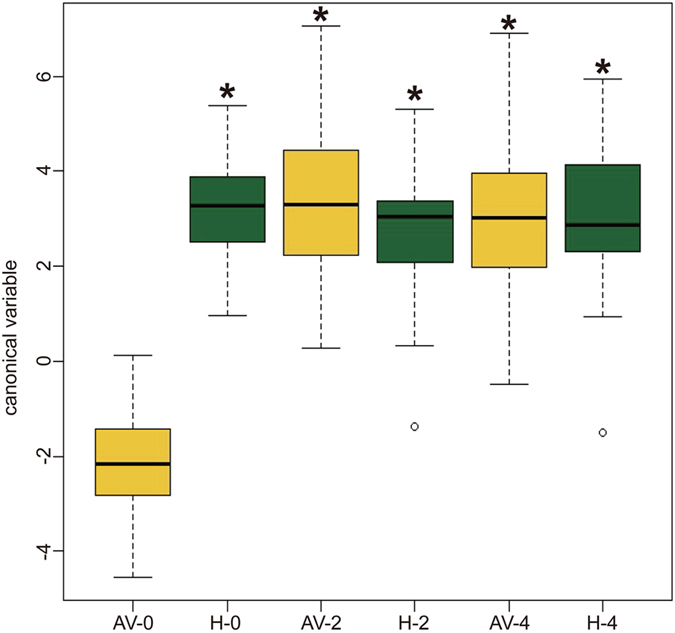
Canonical variable analysis of vaginal bacterial communities in healthy women at 0, 2, and 4 weeks (H-0, H-2, and H-4) compared to each other and the vaginal communities of women with AV at 0, 2, and 4 weeks (AV-0, AV-2, and AV-4). Asterisks indicate communities that were significantly different (P < 0.05) than those of AV-0 (AV patients at time zero).

Changes in the composition of vaginal microbiota in healthy post-menopausal women over time were visualized in interpolated bar plots of phylotype relative abundance (Fig. 2 and Supplementary Fig. S1). The species composition of vaginal communities in healthy post-menopausal women were rather persistent over time despite differences in their composition among individuals (Fig. 3). This was also reflected in the Shannon diversity and equitability (evenness) indices that did not vary significantly over the 4-week study period (Table 2). The stability was exemplified by the vaginal community of subject H-13, which was dominated by *Lactobacillus* and exhibited only minor changes over the four-week study period and all three samples remained in cluster I. Similarly, the communities of subject H-12 (dominated by *Streptococcus*, cluster II) and H-17 (that exhibited co-dominance of several bacterial taxa, cluster III) remained steady during the observation period (Fig. 2B,C) without substantial changes in either the kinds or relative abundances of bacterial taxa present. In contrast, the relative abundances of bacterial taxa in the vaginal community of subject H-4 (cluster III, Fig. 2D) varied at the three time points but the overall composition remained about the same.

### Differences in vaginal microbiota between post-menopausal women with and without atrophic vaginitis

Prior to low-dose estrogen therapy the vaginal communities of women with AV differed markedly from those of healthy women in terms of the relative abundances of bacterial taxa (Table 3), especially with regard to the relative abundances of *Lactobacillus* and *Gardnerella*. Although species of *Lactobacillus* were common in women with AV (80% of subjects), they were less abundant and on average constituted only 11.2% of these communities. This contrasts with the vaginal communities of healthy women wherein lactobacilli were significantly more abundant (*p* < 0.0001) and on average constituted 53.2% of vaginal communities. Conversely, *Gardnerella* was significantly more abundant than *Lactobacillus* in the vaginal communities of subjects with AV (*p* < 0.0001), while also richer in abundance as compared to those of healthy women (41.7% versus 16.7%, *p* < 0.0001).

**Figure 4:**
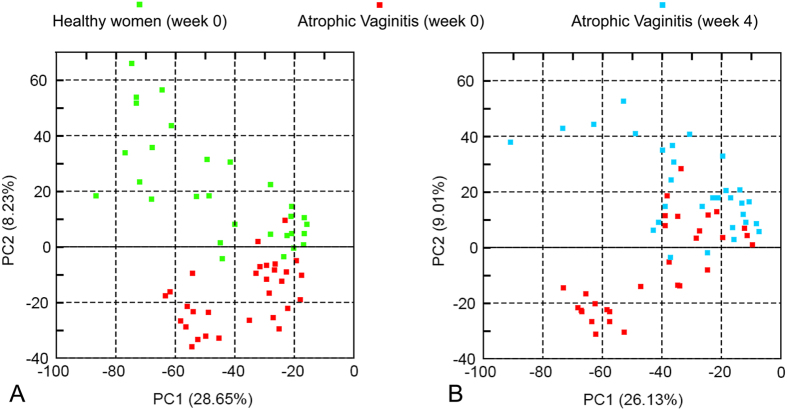
Relationships among vaginal bacterial communities visualized by principal component analysis (PCA). Each point corresponds to a single subject at a single time point and was colored according to group membership and time of sampling. The percentages given in the axis labels wee the proportion of variation explained by that principal component. (**A**) Comparison of vaginal bacterial communities of healthy post-menopausal women at week 0 (green dots) to those of women with atrophic vaginitis at week 0 (red dots). (**B**) Comparison of vaginal bacterial communities of women with atrophic vaginitis at week 0 (red dot) to those in the AV group after 4 weeks of estrogen therapy (blue dots).

Using canonical variable analysis (Fig. 3) we compared the vaginal microbiota profiles of subjects in the H and AV groups. The first canonical variable, which is a linear function of the transformed species proportions, best separated women in these groups at the time of enrollment. At the first sampling time the mean canonical variables of the two groups were significantly different (F = 233.5, P < 0.0001, df = 1, 114, Fig. 3). Differences in the composition of vaginal bacterial communities of healthy women and women with AV at the time of enrollment were visualized by principal component analysis (PCA) (Fig. 4A). However, the overall diversity and evenness of bacterial communities in these two groups were not significantly different between healthy women and those with AV (Table 2).

### Vaginal clinical and microbiota response to low-dose estrogen treatment

AV patients indicated a significant change in their condition when treated with low-doses of estrogen, which was consistent with previous results^9^,^10^. In response to treatments the serum concentration of estradiol increased from an average of 42.05 ± 7.68 pmol/L prior to treatment to an average of 168.07 ± 8.30 pmol/L at week 2 with virtually no further increase between weeks 2 and 4 (Table 4). These changes in serum estradiol levels were accompanied by progressive increases in the VMI and decreases in both symptom scores and vaginal pH (Table 4). The positive effects of estrogen therapy were quite evident after two weeks of treatment. In subjects with AV the mean VMI was 28.1 at the time of enrollment, but increased to 39.3 by week 2 and reached 55.4 by week 4. These changes in VMI were paralleled by decreases in vaginal pH, which was initially 6.6 (on average) at the time of enrollment but decreased to 5.4 and 5.0 after two and four weeks of therapy, respectively. It is not known if further decreases in pH would have occurred with continued therapy.

Low-dose estrogen therapy also had marked effects on the composition of vaginal microbiota. When displayed by PCA, the majority of samples from women in the AV group at week 0 and week 4 were clearly assembled in different regions, indicated that the communities of individuals significantly changed when the host was treated with estrogen (Fig. 4B). A repeated measures ANOVA on the canonical variable for differences between groups and across time showed a significant interaction between group and time (F = 188.7, df = 2, 114, P < 0.0001). Simple effects tests to investigate group differences at each time point showed a trend where the highly significant group difference at time zero (described above) became not quite significant after two weeks and then not significant after four weeks (F = 233.5, P < 0.0001; F = 3.29, P = 0.072; F = 0.28, P = 0.60, respectively, with df = 1, 114 for all three tests). The box plots in Fig. 3 illustrate this pattern wherein communities in most women of the AV group became similar to those in the H group after estrogen treatment. Moreover, decreases of Shannon diversity and Shannon equitability indices were observed (Table 2) indicating that the observed community diversity appeared to decrease in response to treatment.

**Figure 5:**
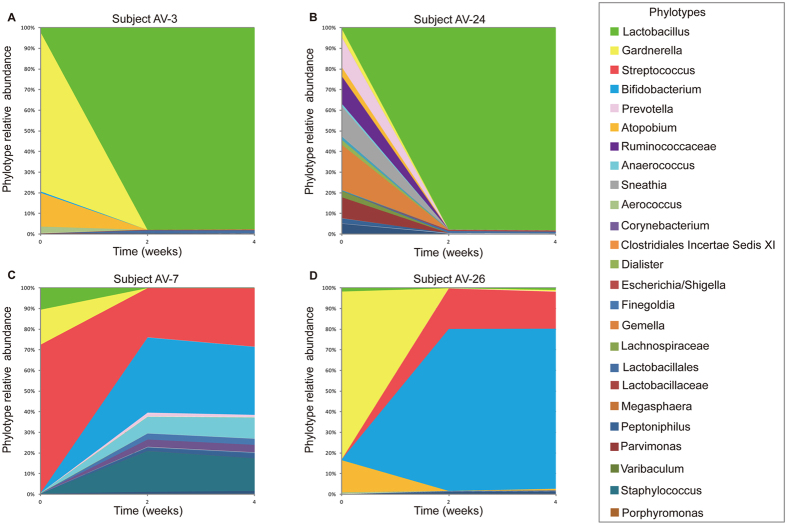
Interpolated bar plots of phylotype relative abundance observed in four subjects selected from the AV group. Color codes for each phylotype represented in the interpolated bar plots are displayed on the right side of the figure. See Supplementary Fig. S2 interpolated bar plots for all subjects.

Concomitant with these changes were profound changes in the species composition of vaginal communities of most women (24/30) treated for AV. The relative proportions of *Lactobacillus* increased dramatically from 11.2 to 71.0% (*p* < 0.0001) and dominated the vaginal communities of 76.7% of treated subjects. This was accompanied by corresponding decreases in the abundances of other prominent bacteria such as *Gardnerella*, *Prevotella,* and *Atopobium* (Table 5). These changes were illustrated in interpolated bar plots of phylotype relative abundances for Subject AV-3 whose vaginal community was dominated by *Gardnerella* before treatment and Subject AV-24 whose vaginal community included a mixture of strictly anaerobic bacteria. In both cases the two communities were transformed to ones dominated by *Lactobacillus* (Fig. 5A,B). Statistical analyses were done to assess whether there was an association between the severity of genital symptoms and specific taxa. We found that *Lactobacillus* was negative correlated (P < 0.0001; Spearman’s test) with symptom scores, whereas *Gardnerella* and *Atopobium* were positively correlated with genital symptoms (Table 5).

Interestingly, the vaginal communities of six women (6/30) treated with estrogen did not transition to a state in which the community was dominated by lactobacilli even though their serum estradiol levels had increased. Instead, there were differing responses among the subjects. Although the communities of four subjects (AV-4, AV-6, AV-7 and AV-9) initially differed from each other, they came to be dominated by *Gardnerella* by week 4 of estrogen therapy (Fig. 5 and Supplementary Fig. S2). In contrast, at the conclusion of estrogen therapy the vaginal communities of two other subjects (AV-11 and AV-26; Supplementary Fig. S2 and Fig. 5) were dominated by *Streptococcus* alone or *Bifidobacterium* and *Streptococcus*, respectively. This showed there can be interpersonal variation among vaginal bacterial communities in response to low dose estrogen therapy.

## Discussion

In this study we found there was a marked and rapid shift in the composition of vaginal communities in most AV patients (24/30) that was accompanied by an alleviation of symptoms. Specifically, *Lactobacillus* came to dominate the vaginal communities by increasing from 11.2 to 71.0% (p < 0.0001) of communities on average in 80% of treated subjects, with a corresponding decrease in the relative proportions of *Gardnerella*, *Atopobium* and *Prevotella.* In most cases the resulting communities closely resembled those found in healthy postmenopausal women. These findings suggest that the emergence of a *Lactobacillus*-dominated vaginal community may be considered as one of the signs of AV treatment success along with the alleviation of genital symptoms, elevated serum estradiol levels and VMI scores. This is consistent with the findings of Hummelen *et al.*^11^ who also found an inverse correlation between the relative abundances of *Lactobacillus* and genital symptom scores although the number of subjects in their study was small (4 AV cases and 6 healthy women).

The results of our study challenge the assertion that to maintain health of the female lower genital tract vaginal communities must be dominated by species of *Lactobacillus* that lower the vaginal pH^12^. We found that while *Lactobacillus spp.* were found in the majority of healthy post-menopausal women it was the dominant species in only 53.2% of vaginal communities that had a mean pH of 6.6 ± 0.2. This is substantially higher than the pH 4.5 threshold that is thought to be necessary for vaginal health and protection in reproductive age women, but concordant with the findings of other studies on postmenopausal women^13^,^14^. Moreover, we found no difference between the vaginal pH of healthy postmenopausal women and women with AV. These findings show that neither high proportions of lactobacilli or a low vaginal pH are required for health in postmenopausal women. The inverse relationship between serum estradiol levels and vaginal pH has long been known^1^,^2^,^3^,^4^ and our findings lend further support to this relationship as we too observed that the vaginal pH of AV patients typically decreased in response to low-dose of estrogen therapy and it remained higher and stable when there were lower levels of serum estradiol. There is increasing evidence that total lactic acid bacterial numbers in the vaginas of post-menopausal women might be 10–100 fold lower than in reproductive age women^15^,^16^ that would sharply reduce the amount of lactic acid production. These data suggest that the vaginal pH of postmenopausal women might best reflect systemic estrogen levels, which are negatively correlated with VMI scores.

Our findings also contravene the widely held view that vaginal communities of post-menopausal women are depauperate of *Lactobacillus*^17^,^18^,^19^,^20^,^21^. Instead our findings are entirely consistent with those who have demonstrated the presence of *lactobacilli* in a fair proportion of postmenopausal women^22^,^23^,^24^,^25^,^26^,^27^. For example, Hillier and Lau *et al.*^27^ found that various species of *Lactobacillus* could be cultured from 50% of postmenopausal women, but the numbers of *Lactobacillus* were 10–100X lower than in reproductive age women. These investigators speculated that the microbiota of postmenopausal women might be similar to those of pre-menarchal girls that also have lower levels of peripheral estrogen. Hickey *et al.*^28^ have recently substantiated this postulate by determining that lactic acid bacteria, primarily *Lactobacillus* spp., were dominant in the microbiota of most girls well before the onset of menarche in the early to middle stages of puberty. In recent years the prominence of *Lactobacillus* in the vaginal microbial communities of some postmenopausal women has been reported^11^,^24^, which is consistent with our findings. This suggests that *Lactobacillus spp. per se* might be more important than pH in sustaining vaginal health after menopause. Aside from the production of lactic acid which itself has antimicrobial properties, *Lactobacillus spp.* produce other antimicrobial compounds such as antibiotics and target-specific bacteriocins^29^ that probably contribute to the antagonistic capacity of *Lactobacillus* and contribute to preventing invasion of the vaginal microbiome by non-indigenous species. Supporting evidence was reported by Rose *et al.*^30^ who showed that certain lactobacilli reduced cytokine secretion in an isolate-specific fashion following TLR stimulation of cultured vaginal epithelial cells and by Bisanz *et al.*^31^ who showed that lactobacilli may regulate epithelial innate immunity in a species-specific manner and by doing so contribute to the microbiome’s ability to effectively exclude pathogenic organisms.

Two bacterial genera, *Gardnerella and Atopobium*, were statistically associated with AV in postmenopausal women. Likewise both of these genera have also been associated with the incidence of **BV** in reproductive age women and shown to have a number of putative virulence determinants^32^. For example, *Gardnerella* has a propensity to form dense biofilms and produce vaginolysin, a member of the cholesterol-dependent family of pore-forming toxins leading to vaginal epithelial cells lysis and death^33^,^34^ while *Atopobium* has been shown to trigger an innate immune response in an *in vitro* model of BV^35^. Others have reported a potential synergism between *Gardnerella* and *Atopobium* because more than 90% of bacteria in biofilms adhering to the vaginal epithelium were comprised of these two genera in women with BV^36^,^37^. However, the roles of these bacteria in AV are unclear since they were also often present in the vaginal communities of healthy post-menopausal women, albeit at somewhat lower proportions. Additionally, these two genera have also occur in the vaginal communities of healthy younger reproductive women and adolescent girls^7^,^38^. The occurrence of *Gardnerella* and *Atopobium* in vaginal microbiomes of healthy and diseased individuals of all ages is a conundrum that might be explained by genetic differences among strains of these species^39^; a proposition that is entirely plausible given the genetic diversity found within both genera.

Brotman *et al.* recently reported that a distinct bacterial community characterized by *Streptococcus* and *Prevotella* with a low relative abundance of *Lactobacillus* is associated with vulvovaginal atrophy^24^. In this study 28 women at different stages of menopause (pre-, peri- and postmenopause) were sampled, but there were only two cases of AV and 14 women with vulvovaginal atrophy. They found no statistically significant associations between clinical variables (menopause stage, signs of vulvovaginal atrophy and vaginal dryness) and any specific species in the communities. Our findings differ from those of Brotman *et al.*^24^ in so far as *Gardnerella* and *Atopobium* were the most frequent and abundant taxa in vaginal communities of women with AV and there was a statistically positive correlation between their prevalence and genital symptom scores. These compelling findings imply that strains of various bacterial species might be associated with the emergence of AV and that new therapeutic targets might emerge as more is learned about the possible causes of AV.

## Methods

### Study Design and Subjects

The individuals enrolled in the study were drawn from outpatients in the Department of Obstetrics and Gynecology of Ruijin Hospital, which is affiliated with the Shanghai Jiaotong University School of Medicine in Shanghai, China. The University’s Institutional Review Board reviewed and approved the project. All patient enrollment occurred between June 2011 and February 2012. After obtaining informed consent participants were screened to confirm eligibility before enrollment. As part of this screening we assessed Vaginal Maturation Index (VMI)^40^, vaginal pH, baseline serum, sexual hormone levels and baseline menopausal quality of life. Women were asked to refrain from sexual activity in the 48 h before the visit.

All methods were carried out in accordance with the guidelines and study protocol approved by the Institutional Review Board and written informed consent was obtained from all subjects that participated in the study. Study subjects with AV (hereafter referred to as the AV Group) met the following criteria: (a) experienced twelve consecutive months of amenorrhea without a period secondary to decreased levels of circulating estrogen caused by depletion of follicles; (b) obvious genital symptoms (e.g. burning, dryness, itching, irritation, soreness and/or bleeding, dyspareunia) with distinct vulvar symptoms that included diminished elasticity, skin turgor, sparse pubic hair, dry labia, dermatoses, lesions, fusion of labia minora, and vaginal symptoms with pale, smooth, lubrication-lacking epithelium or inflammation with patchy erythema, petechiae, increased friability; (c) a positive result on microscopic examination of 5% or less superficial vaginal cells and increased vaginal pH (>5.0); (d) increased follicle stimulating hormone (FSH) concentrations (40 IU/L or more); (e) decreased serum estradiol concentrations (20 pg/mL or less); (f) an endometrial thickness of 5 mm or less as determined by transvaginal ultrasonography, (g) a body mass index between 18.0 and 35.0 kg/m^2^; (h) did not smoke or use tobacco or nicotine; and (i) did not have trichomoniasis or candidiasis. A group of healthy post-menopausal women that did not meet these criteria and had no other preexisting condition were also enrolled and referred to as the H group.

Postmenopausal women were excluded from the study if they had used any antibiotics or antimycotics, vaginal medications, vaginal suppositories, douches, feminine sprays, or genital wipes in the past 30 days, or if they had taken any estrogen products 6 months prior to screening visit (week 0). Other exclusion criteria were systemic diseases or chronic conditions (diabetes mellitus, immunological disease), use of immunosuppressive drugs, exogenous corticosteroids, chemotherapy, or having impaired bladder emptying or pelvic-floor dysfunction causing urinary incontinence, genital prolapse, cystocele, or a history of urogenital surgery^24^.

After enrollment, subjects in the AV Group received low-dose (0.3 mg) conjugated estrogens (Premarin, Wyeth) orally once per day for a 4-week period.

### Sample collection and clinical exams

A physician used the Elution-swab system (Copan) to collect vaginal swab samples from subjects in the AV Group and the H Group at the initial visit (week 0) as well as 2 and 4 weeks later. All swabs were stored in 1 ml of Amies transport medium (Copan) and frozen upright on dry ice until they could be transported to the laboratory, where they were stored at −80 °C. These were used for analysis of bacterial community composition.

During these visits VMI, vaginal pH, serum estradiol and menopausal quality of life were appraised. To determine the VMI of the vaginal mucosa cytology specimens were collected at enrollment (week 0) and at each treatment visit (weeks 2 & 4). The collected cells and mucus were mixed in a fixative solution to form a cell suspension, which was viewed microscopically. The number of superficial, intermediate, and parabasal cells were counted and the percentages of each cell type were calculated. These percentages were used to determine the VMI, which is calculated as follows: VMI = (% intermediate cells × 0.5) + % superficial cells. During the same visit vaginal pH was measured by inserting pH paper into the vagina and comparing the color change result with the manufacturer’s color chart (pHEM-ALERT^®^, Gynex Corporation, Redmond, WA). The serum concentrations of sexual hormones were assessed using electro-chemiluminescence immunoassays (Elecsys 1010 Analyzer, Roche). Lastly, each participant completed a self-assessment of symptoms score which was rated as none, mild, moderate or severe^11^ to assess the severity of genital symptoms such as vaginal dryness, irritation or itching, pain during urination not associated with infection, vaginal soreness, pain during sexual intercourse and bleeding after sexual intercourse.

### Classification of bacterial populations based on sequences of the V1-V3 region of bacterial 16S rRNA genes

All methods and algorithms used to gather and analyze DNA sequence data are provided in Supplementary Methods. In brief, a validated method^7^ was used to extract total genomic DNA (gDNA) from vaginal swabs using the QIAamp DNA Mini Kit (Qiagen, Hilden, GER). The variable V1-V3 regions of 16S rRNA genes in each gDNA sample were amplified in two rounds of PCR with dual barcode indexing using the PCR primer sequences shown in Supplementary Table S1. The final amplicons were sequenced using an Illumina MiSeq platform (Illumina, San Diego, CA, USA) and the RDP Bayesian classifier was used to assign clipped and concatenated sequences to phylotypes (RDP 2.5; http://rdp.cme.msu.edu). For constructing phylotype abundance tables we used a simple heuristic rule: to be included in the table a phylotype had to either: (a) be present in more than one sample at an abundance of 1% or more, or (b) constitute more than 5% of a single community. Phylotypes that did not meet either of these criteria were aggregated into an “Other” category.

## Additional Information

**How to cite this article**: Shen, J. *et al.* Effects of low dose estrogen therapy on the vaginal microbiomes of women with atrophic vaginitis. *Sci. Rep.* **6**, 24380; doi: 10.1038/srep24380 (2016).

## Supplementary Material

Supplementary Information

Supplementary Dataset

## Figures and Tables

**Table 1:** Baseline characteristics of subjects in H and AV groups^a^.

	H group	AV group
Characteristic	(n = 29)	(n = 30)
Age (year)	55.6 ± 2.6	55.8 ± 3.2
Time since menopause (month)	61.8 ± 26.5	64.6 ± 30.6
Body mass index	26.5 ± 0.8	26.5 ± 0.6
Systolic blood pressure (mmHg)	126.2 ± 6.6	126.0 ± 6.7
Diastolic pressure (mmHg)	72.6 ± 8.9	75.3 ± 8.4
Pulse rate (bpm)	75.4 ± 4.1	77.3 ± 4.2
Serum estradiol concentration (pmol/L)	44.8 ± 8.4	42.0 ± 7.7
Vaginal maturation index	29.2 ± 10.7	28.1 ± 11.5
Vaginal pH	6.6 ± 0.2	6.6 ± 0.2

^a^No significant differences between groups for any characteristic. Values are means ± standard error.

**Table 2:** Comparison of community diversity between groups and visits.

Diversity index	H group			AV (H) group		
	Week 0	Week 2	Week 4	Week 0	Week 2	Week 4
Shannon diversity	1.04 ± 0.73	1.03 ± 0.70	1.05 ± 0.67^a^	1.12 ± 0.63^b^	0.60 ± 0.578	0.61 ± 0.58^c^
Shannon equitability	0.29 ± 0.18	0.28 ± 0.16	0.28 ± 0.16^a^	0.36 ± 0.17^b^	0.17 ± 0.14	0.174 ± 0.14^c^

^a^In the H group there was no significant difference (*p* >0.05) in the Shannon diversity and equitability indices for samples collected in weeks 0, 2 and 4.

^b^There was no significant difference between AV Group before treatment (week 0) and N Group (weeks 0, 2 and 4) in Shannon diversity index and equitability (p >0.05).

^c^In the AV group the Shannon diversity and equitability indices in week 0 (before treatment) were significantly higher than those in weeks 2 and 4 (during and after treatment). There was no significant difference in these indices between week 2 and 4 of the AV group.

**Table 3:** Proportions of five prominent genera in the vaginal microbiota of healthy women (H Group, week 0) and women with atrophic vaginitis before estrogen treatment (AV Group, week 0).

Phylotype	H group	AV group	p^a^
	Week 0	Week 0	
*Anaerococcus*	1.8 ± 4.0	0.4 ± 0.8	>0.05
*Atopobium*	2.7 ± 6.9	4.8 ± 5.5	>0.05
*Gardnerella*	16.7 ± 21.7	41.7 ± 31.0	<0.0001^b^^,^^c^
*Lactobacillus*	53.2 ± 40.3	11.2 ± 16.4	<0.0001^d^
*Prevotella*	7.4 ± 13.3	6.0 ± 10.3	>0.05

^a^Student’s t-test was used to assess the statistical significance of differences in the relative proportions of species.

^b^*Gardnerella* was significantly more abundant in women of the AV Group as compared to women in the H Group (<0.0001).

^c^In women of the AV group *Gardnerella* was significantly more abundant than *Lactobacillus* (<0.0001).

^d^*Lactobacillus* was significantly more abundant in women of the H Group as compared to women in the AV Group (<0.0001).

**Table 4:** Clinical response of atrophic vaginitis to short term low-dose estrogen treatment.

Clinical index	Week 0	Week 2	Week 4
Serum estradiol (pmol/L)	42.05 ± 7.68	168.07 ± 8.30^a^	171.48 ± 6.98^b^
Vaginal maturation index	28.07 ± 11.51	39.30 ± 10.75^a^	55.37 ± 7.38^b^
Symptom score	3.93 ± 0.25	1.50 ± 0.63^a^	1.00 ± 0.00^b^
Vaginal pH	6.64 ± 0.20	5.41 ± 0.35^a^	4.95 ± 0.32^b^

^a^The serum estradiol concentration and VMI were significantly higher in week 2 as compared to week 0 (p < 0.0001), while symptom score and vaginal pH in week 2 was significantly lower than in week 0 (p < 0.0001).

^b^The serum estradiol concentration and VMI were significantly higher in week 4 as compared to week week 0 (p < 0.001), while symptom score and vaginal pH in week 4 was significantly lower than that in week 2 and week 0 (p < 0.0001).

**Table 5:** Comparison of relative proportions of five prominent genera in vaginal microbiota of women with AV before and after estrogen treatment.

Phylotype	Week 0	Week 4	*p*^a^
*Anaerococcus*	0.004 ± 0.008	0.019 ± 0.064	>0.05
*Atopobium*	0.048 ± 0.055	0.003 ± 0.012	<0.0001^b^
*Gardnerella*	0.417 ± 0.310	0.096 ± 0.234	<0.0001^b^
*Lactobacillus*	0.112 ± 0.164	0.710 ± 0.375	<0.0001^c^
*Prevotella*	0.060 ± 0.103	0.007 ± 0.014	<0.01^b^

^a^Student’s t-test was used to assess the statistical significance of differences in the relative proportions of species during short term low-dose estrogen treatment.

^b^The relative proportions of *Gardnerella* (p < 0.0001), *Prevotella* (p < 0.01), and *Atopobium* (p < 0.0001) in week 4 were significantly less than in week 0.

^c^The relative proportion of *Lactobacillus* in week 4 was significantly greater than in week 0 (p < 0.0001).
