# Erratum: Effects of low dose estrogen therapy on the vaginal microbiomes of women with atrophic vaginitis

**DOI:** 10.1038/srep34119

**Published:** 2016-11-29

**Authors:** Jian Shen, Ning Song, Christopher J. Williams, Celeste J. Brown, Zheng Yan, Chen Xu, Larry J. Forney

Scientific Reports 6: Article number: 2438010.1038/srep24380; published online: 04 22 2016; updated: 11 29 2016

The original version of this Article contained errors in Affiliations 3, 4 and 5 which were incorrectly given as ‘Departments of Statistical Science, University of Idaho, Moscow, ID, 83844, Russia’, ‘Biological Sciences, University of Idaho, Moscow, ID, 83844, Russia’ and ‘Institute for Bioinformatics and Evolutionary Studies (IBEST), University of Idaho, Moscow, ID, 83844, Russia’ respectively. The correct Affiliations are listed below:

Affiliation 3

Department of Statistics, University of Idaho, Moscow, ID, 83844, USA.

Affiliation 4

Department of Biological Sciences, University of Idaho, Moscow, ID, 83844, USA.

Affiliation 5

Institute for Bioinformatics and Evolutionary Studies (IBEST), University of Idaho, Moscow, ID, 83844, USA.

These errors have now been corrected in the PDF and HTML versions of this Article.

