# Supplementary material for: Effects of low dose estrogen therapy on the vaginal microbiomes of women with atrophic vaginitis
Source: Sci Rep. 2016 Apr 22;6:24380. doi: 10.1038/srep24380 (PMC4840317; doi:10.1038/srep24380)
Supplement: Supplementary Information [file srep24380-s1.doc]

# Supplementary Information

# Effects of low dose estrogen therapy on the

# vaginal microbiomes of women with atrophic vaginitis

Jian Shen, Ning Song, Christopher J. Williams,

Celeste Brown, Chen Xu, Larry J. Forney

# Supplementary Methods

## Whole-Genomic DNA Extraction from Vaginal Swabs

All archived vaginal swab specimens were thawed on ice and then vortexed for 5 minutes to suspend the cells. A 0.5 ml-aliquot was transferred to a sterile 2.0 mL tube with cell lysis buffer composed of 50 μl lysozyme (10 mg/ml, Sigma-Aldrich), 6 μl mutanolysin (25 KU/ml; Sigma-Aldrich, St. Louis, MO, USA), and 3 μl lysostaphin (4000 U/ml, Sigma-Aldrich) and 41 μl of TE50 buffer (10 mM Tris·HCl and 50 mM EDTA, pH 8.0). After 1 hour of incubation at 37°C, 600 mg of 0.1-mm-diameter zirconia/silica beads (BioSpec, Bartlesville, OK, USA) were added to the mixture and cells were mechanically disrupted using Mini-BeadBeater-96 (BioSpec) at 2100 rpm for 1 minute. Further isolation and purification of the total genomic DNA from crude lysates were processed using QIAamp DNA Mini Kit (Qiagen, Hilden, GER) according to the manufacturer’s recommendation except the DNA was eluted into two separate tubes using two 100μl aliquots of AE buffer (10 mM Tris-HCl, 0.5 mM EDTA; pH 9.0). A PicoGreen assay was used to quantify genomic DNA in each sample (Invitrogen, Carlsbad, CA, USA). Fluorescence was determined using a SynergyTM HT Multi-Mode Microplate Reader (BioTek, Winooski, VT, USA) at an excitation wavelength of 485 nm and emission wavelength of 528 nm^1^.

## PCR amplification and sequencing of the V1-V3 region of bacterial 16S rRNA genes

Amplicons were produced at the University of Idaho using de-identified samples and the work done was exempt from IRB oversight.

The variable V1-V3 regions of 16S rRNA genes in each sample were amplified in two rounds of PCR with dual barcode indexing prior to analysis on an Illumina MiSeq platform (Illumina, San Diego, CA, USA). The first PCR round amplified the target specific regions in 16S rRNA genes (E. coli positions 27F-534R), while the second attached sample specific barcodes and Illumina sequencing adapters. The PCR primer sequences used are shown in Table S1.

Using the universal 16S rRNA primers 27F and 534R the V1-V3 regions of 16S rRNA genes were amplified in 96-well microtiter plates using AmpliTag Gold DNA polymerase (Applied Biosystems) and 100ng of template DNA in a total reaction volume of 50μl. The first round of PCR was run in a PTC-100 thermal controller (MJ Research, St. Bruno, Quebec, CAN) using the following cycling parameters: 2 min of denaturation at 95°C, followed by 20 cycles of 1 min at 95°C (denaturing), 1 min at 51 °C (annealing), and 1 min at 72 °C (elongation), with a final extension at 72°C for 10 min. The presence of amplicons was confirmed by agarose gel electrophoresis and staining with SYBRGreen. The second PCR was run in a total reaction volume of 20μl using the following parameters: 10 min of denaturation at 95°C, followed by 10 cycles of 15 s at 95°C (denaturing), 30 s at 51 °C (annealing), and 1 min at 72 °C (elongation), with a final extension at 72 °C for 3 min. Negative controls without a template were included for each primer pair. The concentrations of amplicons were quantified by fluorometry (GeminiXPS, Molecular Devices, Sunnyvale, CA, USA) using PicoGreen, then equimolar amounts (100 ng) of the PCR amplicons were pooled in a single tube. Short DNA fragments and amplification primers were removed from the pool amplicons using AMPure beads (Beckman-Coulter, Indianapolis, IN, USA), and then the purified amplicons were recovered from a 1% agarose gel followed by a second size selection with AMPure beads. The resulting amplicon pool was amplified by PCR using Illumina adaptor specific primers and the PCR product was analyzed on a DNA 1000 chip for the Agilent 2100 Bioanalyzer (Agilent Technologies, Santa Clara, CA, USA). When the entire purification procedure was completed, and no short fragments were observed after PCR, the final amplicon pool was then quantified using the KAPA Illumina library quantification kit (KAPA Biosciences, Wilmington, MA, USA) and the Applied Biosystems StepOne plus real-time PCR system. The de-identified amplicons were sequenced by the Genomics Resources Core of the Institute for Bioinformatics and Evolutionary Studies (IBEST) at the University of Idaho using an Illumina MiSeq platform and a 250bp paired-end protocol (Illumina, Inc., San Diego, CA) with custom sequencing primers (see table of primers) and 10% phiX DNA to increase sequence diversity.

## Read quality control, sequence analysis and taxonomic assignments

Raw unclipped DNA sequence reads from the Illumina platform were cleaned, assigned and filtered in the following manner. Raw FASTQ files for analyzed for barcode assignment (Read 2 and 3, from the Illumina 4 read protocol) allowing for one mismatch. Amplicon primer sequences in Read 1 and 4 were identified using Cross Match (version 1.080806, parameters: min matches=8, min score=16) from the phred/phrap/consed application suite. Cross Match alignment information was then read into R and processed to identify alignment quality, directionality, barcode assignment, and read clip points. Base quality clipping was performed using the application Lucy (version 1.20p, parameters: max average error=0.002, max error at ends=0.002). We then aligned the clipped reads to the SILVA bacterial sequence database http://www.arb-silva.de using mothur (www.mothur.org/‎; version 1.27). Alignment end points were identified and used in subsequent filtering. Sequence reads were filtered to only those that met the following criteria: (a) sequences were at least 100 bp in length; (b) max hamming distance of barcode = 1; (c) maximum number of matching error to forward primer sequences = 2; (d) had <2 ambiguous bases (Ns); (e) alignment to the SILVA bacterial database was within 75 bp of the expected alignment start and stop position; and (f) read alignment started within the first 5 bp and extended through read to within the final 5 bp. The RDP Bayesian classifier was used to assign clipped and concatenated (Reads 1 and 4) sequences to phylotypes (RDP 2.5; http://rdp.cme.msu.edu). Reads were assigned to the first RDP level with a bootstrap score >=50. The proportions of various phylotypes in each sample were then calculated. The depth of coverage for each community was sufficient to detect taxa that constituted ≈0.1% of a community.

## Statistical analysis

Similarities in the composition of vaginal bacterial communities were assessed by computing Ward’s linkage and Hellinger distance and complete hierarchical clustering using R ^1^. The resulting dendrogram reflects the degree of dissimilarity among samples in terms of relative microbial species abundances. Additionally, Spearman’s correlation coefficient proﬁle between communities were calculated and clustering was done with the use of complete linkage in which the maximum distance between two clusters is computed ^1^. Heatmaps were constructed using the TM4 (Version: 4.8) ^2^.

Prior to performing analyses of vaginal bacterial community data to investigate group differences, the proportions of taxa were transformed to log-ratios relative to their geometric mean ^3^:

f(pij) = log[(pij +K)/gmj],

where f(pij) is the transformed proportion for the ith taxon in the jth subject, and gmj is the geometric mean of the set of pij+K values for the jth subject. To avoid problems with observed proportions equal to zero, a small positive constant K was added to each proportion ^3^.

To investigate associations between community composition and differences between the two groups at the first time point, a stepwise discriminant analysis was performed using the log-ratio-transformed proportions for the most abundant 35 species. The subset of species selected was used to create a canonical variable (the best linear combination of species transformed proportions) to separate the two groups at the first time point. This same canonical variable was also calculated for each subject at the other two time points to investigate how the group differences changes over time after treatment. The change over time of the canonical variable between groups was analyzed using a repeated measures analysis of variance with time (3 visits per person) as the repeated measure and the treatment groups as the between-subjects factor. A comparison of different covariance structures showed that a first-order autoregressive covariance structure fit best and was used in the repeated measures analysis. Significant interaction between group and time was further investigated by performing simple effect tests for group differences at each time. Discriminant analyses and repeated measures analyses were conducted in SAS (Version 9.3; SAS Corp., NC, USA).

Principal components analysis (PCA) was used to attribute the variability of samples in the data to a reduced set of variables termed principal components (PC) and the first two PCs were used to map each sample in a two dimensional space. Vaginal communities in healthy postmenopausal women and atrophic vaginitis patients (before and after estrogen treatment) were compared by PCA analysis.

The Shannon diversity index (H) was used to estimate species diversity in vaginal communities. The index is defined as H= -Σ pi log2(pi) where pi denotes the proportion of species i in each sample. To estimate evenness we calculated the Shannon’s equitability (EH) index using the formula EH= H/lnS where S is total number of species in a community ^4^.

Student's t-test was used to assess the statistical significance of differences in baseline characteristics, clinical index, the relative abundances of species, Shannon diversity and equitability indices.

## References

1. Ravel, J., et al. Vaginal microbiome of reproductive-age women. Proc. Natl. Acad. Sci. 108S1**,**4680-4687 (2011).
2. Saeed, A.I. et al. TM4: a free, open-source system for microarray data management and analysis. Biotechniques 34**,** 374-378 (2003).
3. Aitchison, J. in The Statistical Analysis of Compositional Data, 416 pp. (The Blackburn Press, 1968).
4. Ling, Z. et al. Molecular analysis of the diversity of vaginal microbiota associated with bacterial vaginosis. BMC Genomics 11**,** 488 (2010).

# Supplementary Figures

Supplementary Figure S1. Interpolated bar graphs that depict the relative abundances of phylotypes in vaginal communities of healthy women (H group) that were sampled at 3 times during the study (week 0, week 2, and week 4).

Supplementary Figure S2. Interpolated bar graphs that depict the relative abundances of phylotypes in vaginal communities of women diagnosed with AV at the time of enrollment (AV group) and subsequently sampled at 3 times during the study (week 0, week 2, and week 4).

# Supplementary Tables

Table S1. Barcoded PCR primers used for the amplification of 16S rRNA genes*.

27F Primer Primer Sequence

27F-YM1 5’ - ACACTGACGACATGGTTCTACAGTAGAGTTTGATCCTGGCTCAG – 3’

27F-YM2 5’ - ACACTGACGACATGGTTCTACACGTAGAGTTTGATCCTGGCTCAG – 3’

27F-YM3 5’ - ACACTGACGACATGGTTCTACAACGTAGAGTTTGATCCTGGCTCAG – 3’

27F-YM4 5’ - ACACTGACGACATGGTTCTACATACGTAGAGTTTGATCCTGGCTCAG – 3’

27F-Bif 5’ - ACACTGACGACATGGTTCTACAGTACGTAGGGTTTGATCCTGGCTCAG – 3’

27F-Bor 5’ - ACACTGACGACATGGTTCTACACGTACGTAGAGTTTGATCCTGGCTCAG – 3’

27F-Chl 5’ - ACACTGACGACATGGTTCTACAACGTACGTAGAATTTGATCTTGGTTCAG– 3’

534R Primer Primer Sequence

534R_1 5’ - TACGGTAGCAGAGACTTGGTCTCCATTACCGCGGCTGCTGG - 3’

534R_2 5’ - TACGGTAGCAGAGACTTGGTCTGCCATTACCGCGGCTGCTGG - 3’

534R_3 5’ - TACGGTAGCAGAGACTTGGTCTTGCCATTACCGCGGCTGCTGG - 3’

534R_4 5’ - TACGGTAGCAGAGACTTGGTCTATGCCATTACCGCGGCTGCTGG - 3’

534R_5 5’ - TACGGTAGCAGAGACTTGGTCTCATGCCATTACCGCGGCTGCTGG - 3’

534R_6 5’ - TACGGTAGCAGAGACTTGGTCTTCATGCCATTACCGCGGCTGCTGG - 3’

534R_7 5’ - TACGGTAGCAGAGACTTGGTCTATCATGCCATTACCGCGGCTGCTGG - 3’

Adapter Primers Primer Sequence

P5 – CS1 5’ - AATGATACGGCGACCACCGAGATCTACACNNNNNNNNACACTGACGACATGGTTCTACA- 3’

P7 – CS2 5’ - CAAGCAGAAGACGGCATACGAGATNNNNNNNNTACGGTAGCAGAGACTTGGTCT- 3’

Sequencing Primers Primer Sequence

FL1 – CS1 5’ - ACACTGACGACATGGTTCTACA- 3’

FL1 – CS2 5’ - TACGGTAGCAGAGACTTGGTCT- 3’

FL2 – CS1rc 5’ - TGTAGAACCATGTCGTCAGTGT- 3’

FL2 – CS2rc 5’ - AGACCAAGTCTCTGCTACCGTA- 3’

***** The underlined sequences are the universal 16S rRNA primers 27F and 534R, which includes seven different 27F primer sequences to capture a broad spectrum of taxa. The bold letters denote the universal sequence tags CS1 and CS2 included in both rounds of PCR primers and the italicized bases are added to the template specific primers to introduce variability of base calls during Illumina sequencing. The adapter primers include the Illumina specific sequences P5 as well as P7 for dual indexing, and the 8-bp barcode is denoted by eight italicized Ns which allow us to simultaneously sequence the amplicons from all samples using relatively few barcoded adapter primers and subsequently assign sequences to the corresponding samples from which they were obtained.
